# Chondrodysplasia, enchondromas and a chest deformity causing severe pulmonary morbidity in a boy with a *PTHLH* duplication: A case report

**DOI:** 10.1016/j.bonr.2021.101067

**Published:** 2021-04-15

**Authors:** Carline E. Tacke, Suzanne W.J. Terheggen-Lagro, Annemieke M. Boot, Astrid S. Plomp, Abeltje M. Polstra, Rick R. van Rijn, Peter A.A. Struijs, Henk van den Berg, Christiaan F. Mooij

**Affiliations:** a Department of Pediatric Endocrinology, Emma Children's Hospital, Amsterdam UMC, University of Amsterdam, Amsterdam, the Netherlands; b Department of Pediatric Pulmonology, Emma Children's Hospital, Amsterdam UMC, University of Amsterdam, Amsterdam, the Netherlands; c Department of Pediatric Endocrinology, Beatrix Children's Hospital, University Medical Center Groningen, University of Groningen, Groningen, the Netherlands; d Department of Clinical Genetics, Amsterdam UMC, University of Amsterdam, Amsterdam, the Netherlands; e Department of Radiology and Nuclear Medicine, Amsterdam UMC, University of Amsterdam, Amsterdam, the Netherlands; f Department of Orthopedic Surgery, Amsterdam UMC, University of Amsterdam, Amsterdam, the Netherlands; g Department of Pediatric Oncology, Emma Children's Hospital, Amsterdam UMC, University of Amsterdam, Amsterdam, the Netherlands

**Keywords:** AP, anteroposterior, CT, computed tomography, DEXA, dual-energy X-ray absorptiometry, IHH, Indian hedgehog, PA, posteroanterior, PTHLH, parathyroid hormone-like hormone, PTHrP, parathyroid hormone related peptide, SDS, standard deviation score, Parathyroid hormone-like hormone, *PTHLH*, Chondrodysplasia, Enchondroma

## Abstract

Parathyroid hormone-like hormone (PTHLH) plays an important role in bone formation. Several skeletal dysplasias have been described that are associated with disruption of PTHLH functioning. Here we report on a new patient with a 898 Kb duplication on chromosome 12p11.22 including the *PTHLH* gene. The boy has multiple skeletal abnormalities including chondrodysplasia, lesions radiographically resembling enchondromas and posterior rib deformities leading to a severe chest deformity. Severe pulmonary symptoms were thought to be caused by limited mobility and secondary sputum evacuation problems due to the chest deformity. Imaging studies during follow-up revealed progression of the number of skeletal lesions over time. This case extends the phenotypic spectrum associated with copy number variation of *PTHLH*.

## 1 Introduction

Parathyroid hormone-like hormone (PTHLH; MIM 168470), also known as parathyroid hormone related peptide (PTHrP), plays an important role in endochondral bone development. PTHLH binds to the parathyroid hormone receptor (PTHR1) in a paracrine way and is responsible for maintaining chondrocytes in an undifferentiated proliferative state. The production of PTHLH is under control of Indian Hedgehog (IHH). Together, IHH and PTHLH form a feedback loop that regulates the timing of chondrocyte differentiation and subsequently endochondral ossification (Vortkamp et al., 1996; Kronenberg, 2006).

Several skeletal dysplasias have been described that are caused by impaired PTHLH-PTHR1 signaling. For example, loss-of-function mutations in *PTHR1* result in a recessive lethal chondrodysplasia, known as Blomstrand chondrodysplasia (Karaplis et al., 1998). Other conditions caused by point mutations, deletions or genomic rearrangements of *PTHLH* or *PTHR1* include brachydactyly type E, Jansen metaphyseal chondrodysplasia, and Eiken syndrome (Klopocki et al., 2010; Thomas-Teinturier et al., 2016; Duchatelet et al., 2005). Skeletal abnormalities, as a result of an increased copy number of *PTHLH*, have also been reported. To date, ten individuals with a duplication encompassing *PTHLH* have been described (Gray et al., 2014; Collinson et al., 2010; Flöttmann et al., 2016). Acro-osteolysis, brachydactyly, bowed long bones and multiple enchondromas were reported in these patients.

Enchondromas are common benign cartilage tumors almost exclusively arising in the metaphyses of long bones and in the small bones of hand and feet. These tumors develop close to the growth plate cartilage where endochondral bone ossification occurs and then extend towards the diaphysis. In childhood, most enchondromas are asymptomatic. Malignant transformation of enchondromas can occur. Reported signs of enchondromas in children include widening of the bone, angular deformity, and limb-length discrepancy. A pathological fracture is in many cases the presenting symptom of an enchondroma.

In this manuscript, we report on a patient with a small duplication of chromosome 12 including *PTHLH*. The boy has multiple skeletal abnormalities including enchondromas, chondrodysplasia, and posterior rib deformities resulting in a severe chest deformity and secondary pulmonary symptoms. This case extends the phenotypic spectrum associated with copy number variation at the PTHLH locus.

## 2 Clinical report

### 2.1 Initial presentation

The boy was born after a normal pregnancy of non-consanguineous parents. His weight at birth was 3960 g (P75). When he was almost two years old, he was seen with a swelling of his right elbow at the emergency department of a local hospital. He had been using his arm normally, but his mother had noticed the swelling and mentioned a limited range of motion of the forearm. There were no evident signs of pain. At physical examination, besides the swelling and limited mobility of his elbow, a remarkable deformity of his chest was observed. The mother had noticed this deformity a few months earlier. Radiography of his arm showed a lesion suspect for a fracture of his right proximal radius. A skeletal survey was performed. Multiple radiological abnormalities were noted. Subsequently he was referred to our tertiary center for further evaluation. Our assessment of radiological findings suggested a skeletal dysplasia.

**Fig. 1:**
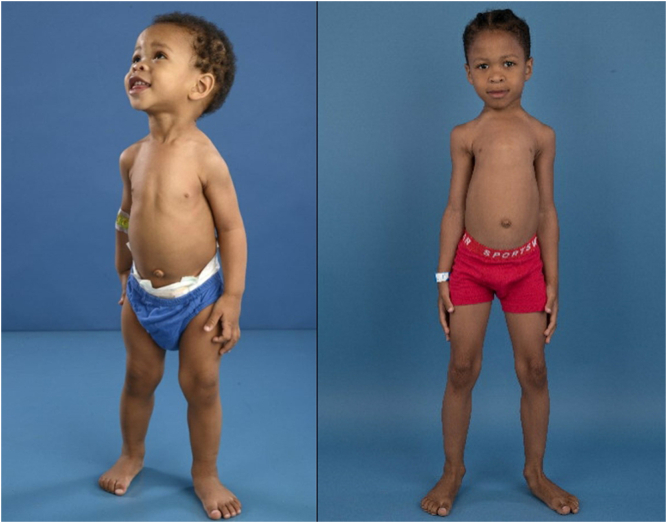
A small and deformed chest in a boy with a *PTHLH* duplication at presentation (almost two years old) and during follow-up (at the age of six years).

**Fig. 2:**
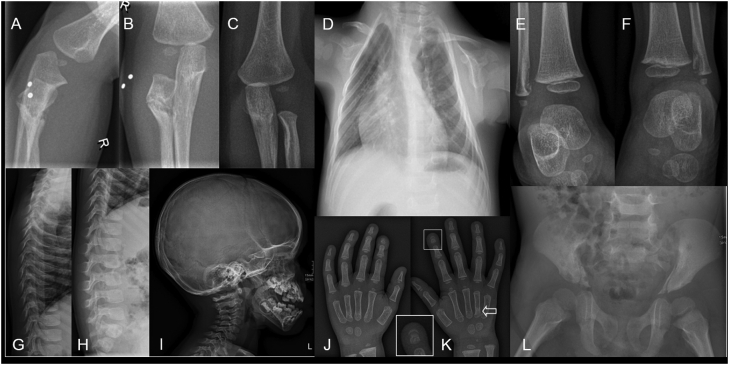
Radiographs made as part of the diagnostic work-up at presentation. A, B, C. Anteroposterior (AP) and lateral radiograph of the right (two radiopaque markers) and AP left elbow. The radiograph of the right elbow was initially interpreted as a fracture of the proximal radius. The radius shows a metaphyseal well-demarcated lucency with local angulation. The AP radiographs shows sclerotic margins of the lesion. The lateral side of the metaphysis of the left radius shows a cortical irregularity and slight bowing. D. Slightly oblique AP chest radiograph shows the deformity of the chest with slender down slanted ribs. E, F. AP radiographs of the ankles shows symmetrical metaphyseal cortical irregularity of the medial side of the fibulas. G, H, I. Normal development of the thoracolumbar spine and skull. J, K. Posteroanterior (PA) radiograph of both hands, there is some sclerosis at the base of the fourth metacarpal of the right hand (arrow). The tuft of the second digit of the right hand is irregular (insert). L. AP pelvis, note the linear irregularities on the inside of the iliac bones. There is slight flaring of the iliac bones and mild dysplasia of the hips.

At initial evaluation in our center, the boy had a short stature (height −1,98 standard deviation score (SDS); target height + 0,10 SDS) with a normal sitting height/height ratio (−0,45 SDS). The deformed, small form of his chest was evident (Fig. 1A). The tips of his index finger were remarkably broad. Dentition was normal and cognitive development appeared to be normal. Laboratory investigations showed no abnormalities, in particular no signs of a disorder in the calcium-phosphate metabolism. The key radiological findings included chondrodysplasia, lesions resembling enchondromas and abnormal rib morphology leading to a severe deformity of his chest. A detailed description of the skeletal abnormalities at the initial presentation is given in Fig. 2.

### 2.2 Genetic investigations

**Fig. 3:**
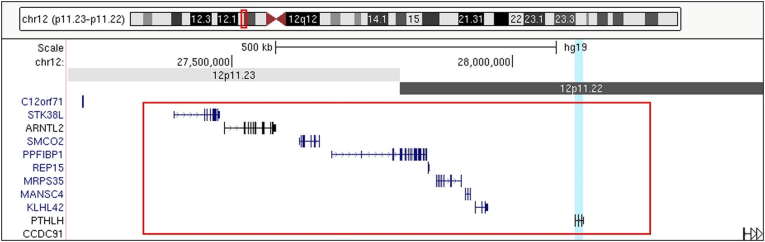
Genomic locus of the duplication region on chromosome 12p11. The duplication is outlined in the red box, containing 9 genes including *PTHLH* highlighted in blue. The other OMIM genes within this region are *STK38L*, *ARNTL2*, *PPFIBP1*, *REP15*, *MRPS35* and *KLHL42*.

The array comparative genomic hybridization (CGH) analysis on a peripheral blood sample, using the Agilent 180 K oligo-array (Amadid 023363; Agilent Technologies, Inc., Palo Alto, CA), showed a duplication of a small part of chromosome 12 (898 Kb): 12p11.23p11.22 (27,337,100-28,234,930). This region contains nine genes including *PTHLH*, the only OMIM morbid gene within the duplication (Fig. 3). The duplication was not present in his non-affected parents.

### 2.3 Skeletal complaints and treatment with bisphosphonates

**Fig. 4:**
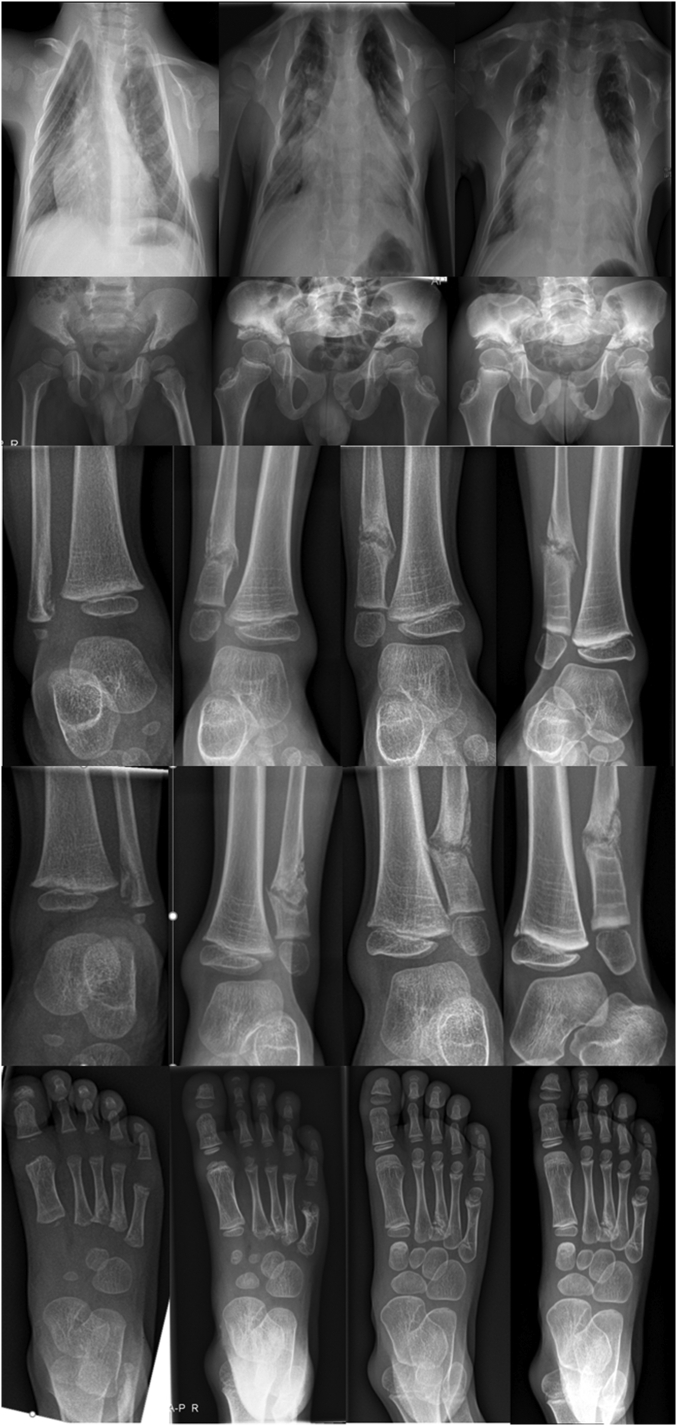
Radiologic findings at presentation and during follow-up. Upper row: Chest development at presentation (1 years 4 months) and during follow-up (3 years 5 months and 4 years 6 months). The chest radiographs shows relatively short down slanted ribs with proximal sclerotic irregularities with at multiple levels interruption of the ribs posteriorly. Over time the chest narrows. Also note the irregular aspect of the medial claviculae. Second row: AP pelvic radiograph at presentation, and 25 and 38 months after first presentation. Note the irregular and dysplastic acetabular development. The irregularities on the inside of the pelvic ring have normalized over time. Note growth resumption lines in the iliac bones and proximal femora as a result of bisphosphonate treatment. Third and fourth row: Radiographs of both ankles at presentation and during follow-up to age 7 years. Imaging shows the development of bilateral symmetric anomalies radiographically resembling enchondromas. As a result of growth the lesions ‘move away’ from the growth plate. Over time the lesions develop into a ‘pseudo-arthrosis’. Note growth resumption lines in the distal tibia and fibula as a result of bisphosphonate treatment. Fifth row: AP radiographs of the feet at presentation and during 5 years of follow-up. Figures show an irregular ossification of the tuft of the first digit of the left foot, irregular ossification of the distal metaphysis of the fifth digit of the right foot, and a well demarcated lucency at the base of the third metatarsal of the right foot. The fifth metatarsal of the right foot shows an irregular distal metaphysis and significant shortening.

During follow-up the boy had multiple skeletal complaints and several imaging studies were performed. These studies revealed ongoing development of new lesions radiographically resembling enchondromas (Fig. 4). A bone biopsy has not been performed in this patient.

**Fig. 5:**
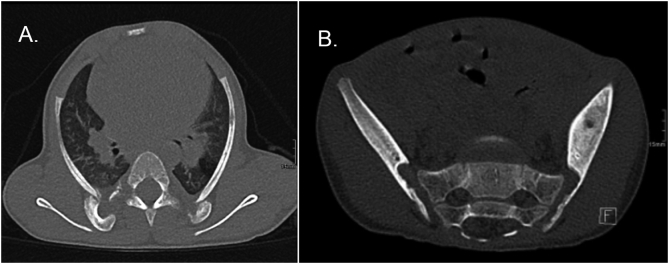
A. CT of the chest (axial oblique reconstruction) 57 months after initial presentation, shows an anomalous development of the proximal part of the ribs with a strong curvature and bilateral ‘pseudoarthrosis’ of the ribs at multiple levels. Due to the posterior anomaly the ribs are relatively short. B. On CT, 32 months after first presentation extreme thinning of the iliac bones at the level of the sacroiliac joint is visible.

Two months after the initial presentation, he complained of pain in his right hip. He was unable to stand and walk. He had no fever or other symptoms. There was no history of any trauma. Radiography showed a deformed pelvis. The pain of the hip disappeared spontaneously over the following months, but 2 years later there was a new episode of pain. Radiography and computed tomography (CT) suggested impaction of the supra-acetabular lesions. On CT there was extreme thinning of the iliac bone at the level of the sacroiliac joint (Fig. 5). Pain subsided after some time. During further follow-up there were two episodes with acute foot complaints; on imaging new lesions were noted (Fig. 4). He was treated twice with a short period of casting and complaints diminished quickly. Deformity of his chest was still clearly present at the age of six years (Fig. 1B).

Treatment with bisphosphonates was started at the age of five years and five months. Before start, a dual-energy X-ray absorptiometry (DEXA) scan had revealed a bone mineral density *Z*-score of −1,3 at the lumbar spine. Start of the treatment was based on the osteopenia on the imaging studies, increased risk of (pathological) fractures in conjunction with bone pain and decreased physical activity. He was treated with zoledronic acid (Zometa®) intravenously every three months with a dose of 0.05 mg/kg (maximum dose 4 mg/day). Since the start of this treatment his physical activity increased and he is walking more stable. A follow-up DEXA scan at the age of 6 years and 8 months showed an increase in bone mineral density at the lumbar spine (*Z*-score 1.0). Subsequently the dose of zoledronic acid was reduced to prevent bisphosphonate-induced osteopetrosis in the unaffected bone.

### 2.4 Pulmonary complications

Based on persistent oxygen need during a lower respiratory tract infection at the age of four years he was seen by the pediatric pulmonologist. He reported to have had recurrent lower respiratory tract infections leading to five hospital admissions within 1.5 years and recurrent treatment with antibiotics. Lung function testing showed abnormalities compatible with a severe restrictive lung disease (predicted forced vital capacity 25%). Chest CT scan showed fibrotic strains in the lower lobes and some air-trapping in the upper lobes. It also showed posterior rib deformities, giving rise to the deformed chest and decreased lung volume (Fig. 5). Immunological screening was normal and heart ultrasound did not reveal signs of pulmonary hypertension. Evaluation by the pediatric cardiologist revealed mild right ventricular dilatation probably due to an increased resistance of the pulmonary vascular bed due to the pulmonary fibrosis and deformed thorax.

His pulmonary complaints were thought to be the result of the chest deformity resulting in limited thoracic mobility and secondary sputum evacuation problems. Supportive treatment was started and consisted of nocturnal oxygen supply, antibiotic prophylaxis and hypertonic saline nebulization to improve sputum evacuation. Polysomnography also revealed nocturnal alveolar hypoventilation and hypercapnia. Nocturnal non-invasive home ventilation resulted in an increase in energy levels during the day and more stable lung disease without additional admittance anymore. There has been no progression of his lung disease under current treatment assessed clinically and by repeated chest CT-scan after 1.5 years.

## 3 Discussion

Here we report on a patient with a 898 Kb duplication on chromosome 12p11.22 encompassing *PTHLH*. The genetic defect resulted in multiple skeletal abnormalities including chondrodysplasia, bone lesions radiographically resembling enchondromas and posterior rib abnormalities causing a severe chest deformity and secondary pulmonary problems. This case illustrates that disruption of *PTHLH* signaling is associated with human disease. *PTHLH* is a key player in endochondral bone development and regulates the proliferation of chondrocytes in developing bone to prevent premature differentiation. The clinical signs and symptoms in our patient provide additional information on the phenotypic spectrum of *PTHLH*-related disorders. This case illustrates that a chest deformity can cause severe secondary pulmonary symptoms in patients with a *PTHLH* duplication. Severe pulmonary symptoms have not been described in literature before in patients with a *PTHLH* duplication. In addition, the follow-up with multiple imaging studies is of specific interest in our patient, and it shows that progression of skeletal lesions over time occurs.

To date, ten patients with a *PTHLH* duplication have been described in literature (Gray et al., 2014; Collinson et al., 2010; Flöttmann et al., 2016). The size of the duplication in these patients differed and included *PTHLH* only up to a maximum of twelve other genes. In 2016, Flöttmann et al. first reported on six patients of a three-generation pedigree with a microduplication containing only *PTHLH* (Flöttmann et al., 2016). These six patients had severe brachydactyly, short humerus and curved radius, but no signs of chondrodysplasia or enchondromas. This is in contrast with the other four previously reported patients (and our patient) with larger duplications, who all had signs of chondrodysplasia and enchondromas. We hypothesize that the skeletal phenotype in patients with *PTHLH* duplications depends on whether (a part of) the regulatory landscape around *PTHLH* is also included in the duplication. This is supported by recent observations in a patient with a 2.802 Mb deletion upstream of *PTHLH* (Deshwar et al., 2019). Although this deletion did not disrupt the copy number of *PTHLH*, the patient had multiple skeletal abnormalities including numerous long bone fractures and metaphyseal changes. The deleted region contained multiple predicted areas of contact with *PTHLH* indicative of putative regulatory sequences (Deshwar et al., 2019). Together, this suggests that not only *PTHLH* but also the genetic region around this gene has a possible influence on the skeletal phenotype. Further research about this interesting genetic region is needed because of a lack of knowledge at this moment.

Our patient has posterior rib deformities causing a chest deformity leading to severe secondary pulmonary symptoms. The deformity leads to decreased thoracic mobility with mucus stasis, and a protracted course of lower respiratory tract infections and local scarring of lung tissue. Supportive treatment consisted of non-invasive ventilation during sleep, antibiotic prophylaxis and airway clearance techniques combined with nebulization of hypertonic saline. With this supportive treatment his pulmonary condition remained stable. Gray et al. previously described rib deformities in two other patients with *PTHLH* duplications (Gray et al., 2014). One patient had a narrow chest, like our patient, with the posterior ends of the ribs sloping downwards anteriorly. In the other patient there was thoracic hypoplasia with irregularity of rib length and shape. In both reported patients, pulmonary symptomatology and function were not described. Based on the clinical course of our patient we suggest that early evaluation of the pulmonary condition is needed. Start of supportive treatment is warranted in patients with a severe chest deformity due to a *PTHLH* duplication to limit pulmonary morbidity as much as possible.

The follow-up of more than five years since diagnosis in this case gives important information about the possible natural course of the disease. Fig. 4 illustrates that radiolucent lesions may develop over time, resulting in a more extensive clinical picture at an older age. Although the exact nature of the lesions is unknown because of the lack of a bone biopsy in our patient, the lesions radiographically resemble enchondromas. In none of the other reported cases with a *PTLH* duplication histopathology has been described.

In our patient treatment with zoledronic acid was started because of the risk of pathological fractures, and the bone pain in conjunction with impaired motoric activity. In only one other patient with a *PTHLH* duplication treatment with bisphosphonates was described, without reporting on its effect. Treatment with zoledronic acid clinically improved his well-being with less pain and increased physical activity.

## 4 Conclusion

In summary, we present the clinical findings of a patient with a rare duplication on chromosome 12p11.22 including *PTHLH*. This report provides additional information on the phenotypic spectrum of *PTHLH*-related disorders. It highlights the potential severe pulmonary complications due to chest deformity, which potentially could be prevented by early start of supportive treatment. To improve the skeletal pain and clinical well-being treatment with bisphosphonates might be beneficial in these patients.

## Funding

This research did not receive any specific grant from funding agencies in the public, commercial, or not-for-profit sectors.

## Transparency document

Transparency document. Image 1

## Declaration of competing interest

The authors declare that they have no known competing financial interests or personal relationships that could have appeared to influence the work reported in this paper.
